# Public and private partnership in disaster risk management

**DOI:** 10.4102/jamba.v8i1.277

**Published:** 2016-09-21

**Authors:** Marino L. Eyerkaufer, Fabiana S. Lima, Mirian B. Gonçalves

**Affiliations:** 1 Department of Accounting Sciences, State University of Santa Catarina, Brazil; 2 Department of Production and Systems Engineering, Federal University of Santa Catarina, Brazil

## Abstract

Private and public partnerships are defended by both guidelines for action and legal frameworks for disaster risk management. The objective of this study is to identify a framework for action that allows joint collaborative partnership between these sectors. The theoretical discussion brings concepts that raise questions that permeate the possibility of partnership based on the new Sendai framework, as well as corporate social responsibility in the value, balance and accountability (VBA) integrative model. The presented framework is compared to the experience of the tornado which occurred in Brazil in the city of Xanxerê (Santa Catarina) in 2015. We came to the conclusion that partnership advance results from paradigm shifts in both sectors, on the one hand, with the development of management mechanisms that clearly define roles and responsibilities of those involved, and, on the other hand, motivation for responsible business conduct.

## Introduction

Intersectoral collaboration is part of the Sendai framework for Disaster Risk Reduction 2015–2030, signed by 187 UN member countries, which deals with a comprehensive preventive approach focused on the people involved with disaster risks, as well as with multiple risk reduction practices and with an inclusive and accessible multiple sector base in order to be efficient and effective (United Nations, International Strategy for Disaster Reduction [UNISDR] 2015).

The Sendai framework also advocates that it is the governament’s responsibility to assume the leadership, regulation and coordination role, in addition to communicating with all the people involved in the design and implementation of policies, plans and regulations. The public and private sectors, the civil society organisations and the academy should work more closely together and create opportunities for collaboration, and integrate disaster risks into businesses’ management practices (UNISDR 2015).

In addition to the Sendai framework, public–private partnership (PPP) is also recommended by the *Brazilian Civil Defence Act* (Brasil, Law no. 12.608/12), and there is also motivation for the internationalisation of corporate social responsibility (CSR).

CSR is perceived by the posture, actions and behaviours of those organisations that voluntarily contribute to social welfare as a continuous process and to the improvement in the relationship with their stakeholders and non-stakeholders (Schwartz & Carroll 2007). CSR is analysed from an implementation of disaster risk management (DRM) perspective.

The concept of partnership derives from the exchange of knowledge, experience and core competency among public and private organisations. The term ‘partnership‘ used in this study refers to the collaboration among public and private organisations, departing, thus, from the concept that guides the long-term contracts between the government and the private initiative, which involves economic interests.

The justification for this study aligns with the search for improved DRM in the communities involving private organisations, initially acting in their own environment and then seeking external collaboration and public organisations’ encouragement regarding strategic management with clear objective definition, which can be realised through PPP actions. Such an approach can help society improve local DRM systems, which act upon life, environment and patrimony protection and, above all, lead to sustainable development.

## Literature review

In this section, we briefly present the elements that underpin the PPP discussion in DRM.

### Public–private partnerships and corporate social responsibility

The discussion on the possibility of PPP stems from a well-known need for greater intersectoral collaboration in DRM processes. Moreover, the current discussion on private businesses’ CSR also increases motivation for training partnerships. PPP is a formal complementary relationship between public and private organisations with a common objective (Koven & Strother 2016).

PPP participants are organisations with different aims. Tachizawa (2014) presents the sociological terminology that distinguishes organisations, namely first, second, and third sector. Whereas the first sector refers to a group of direct and indirect management governmental organisations that provide public service, the second sector refers to the market, that is, private organisations which are motivated by profit and return on investment. Finally, the third sector refers to non-profit, public purpose private civil society organisations (known as non-governmental organisations [NGO]’s). This study focuses on first and second sector PPP in DRM.

Tomasini and Van Wassenhose (2009) point out that disasters have forced private organisations to reexamine their role and consider humanitarian activities in terms of strategy and social responsibility. By becoming better corporate citizens these companies believe that they can be of benefit to both their business and society, despite the risks involved. On the other hand, humanitarian organisations recognise that the private sector can help with resources and competencies, as well as create opportunities to improve their impact on society by means of responsible actions, strengthened by partnership, that further allow the exchange of better practices and knowledge. When successful, PPP has the potential to explore both parties’ core competencies, optimising the DRM process (Tomasini & Van Wassenhose 2009).

Discussing PPP is even more important because of disaster statistics that show an increase in frequency and intensity, which is a worrying trend to organisations as it is a threat to lives, subsistence and health, and people’s, companies’, communities’ and countries’ economic, physical, social, cultural and environmental assets. The objective of the study is to identify actions that allow the implementation of PPPs in DRM.

Society incurs substantial economic losses caused by natural or man-made catastrophes or disasters. Therefore, governments need to protect themselves against the financial impact of such events. Authors such as Margulescu and Margulescu (2013), Rakesh and Sriparna (2014) and Tomasini and Van Wassenhose (2009) suggest that new forms of cooperation between private and public sectors can help countries’ finance disaster risks.

From a general point of view, CSR is directly related to PPP modus operandi because, considering that CSR is the management defined by the company’s ethical and transparent relation with all players involved and by the setting up of corporate targets which are compatible with society’s sustainable development (Instituto Ethos De Empresas E Responsabilidade Social [ETHOS] 2006), PPP-defined modus operandi directly incorporates such a concept and, therefore, CSR is one of the topics of this modus operandi.

The increase in the number of natural disasters has forced private companies to reexamine their roles and also consider humanitarian activities in terms of strategy and social responsibility. By becoming better corporate citizens these companies believe that they can be of benefit to both their business and society, despite the risks involved (Tomasini & Van Wassenhose 2009). They must consider why and how to adjust their best practices based on needs, potentially reducing response time, which can make decision-making more agile, facilitate communication and transform need into action.

The Organisation for Economic Co-Operation and Development (OECD) (2005) sees as a trend the state continuous disengagement from direct provision of services, as well as the intensification of the regulatory role. For the organisation, improved observance and transparency, better results with emphasis on planning and accountability and result control are some of the challenges for public management. According to Marini and Martins (2004), good social governance depends on the balance between the state, the market and non-profit organisations’ capability and power.

Therefore, PPP, as an interrelation between public and private organisations, is a possibility for the development and implementation of a more responsible political project (Auzzir, Haigh & Amaratunga 2014). Auzzir *et al*. (2014) underline the following PPP characteristics presented in the specialised literature: (1) mutual coordination, (2) the need for risk and profit sharing, which can occur either as financial profit or social benefits, (3) partners’ willingness to improve the cooperation process, for example, a group of informal project, cooperatives or other forms of hybrid organisation.

PPP benefits are widely discussed by many authors, such as Williamson (1996), McQuaid (2000) and Savas (2000). As shown by Auzzir *et al*. (2014), PPP increases efficiency and reduces costs. Each player’s knowledge and skills are fully utilised, and each of the players involved has an incentive to improve their efforts towards increasing the value of the product or service that is being delivered (Huxham & Vangen 2013; Johannessen *et al*. 2013; Parker & Vaidya 2001 cited in Auzzir *et al*. 2014). The new Sendai framework (UNISDR 2015) shows adherence to the public and private theme when it refers to intersectoral and community collaboration as a principle for the disaster risk reduction.

It is in such a scenario that Mandell (2001) ratifies the importance of PPP in the elaboration and implementation of political projects that focus on social problems. Beratan (2007) defines disaster management as a complex social problem due to the frequency of disasters and substantial damage caused to communities.

**FIGURE 1:**
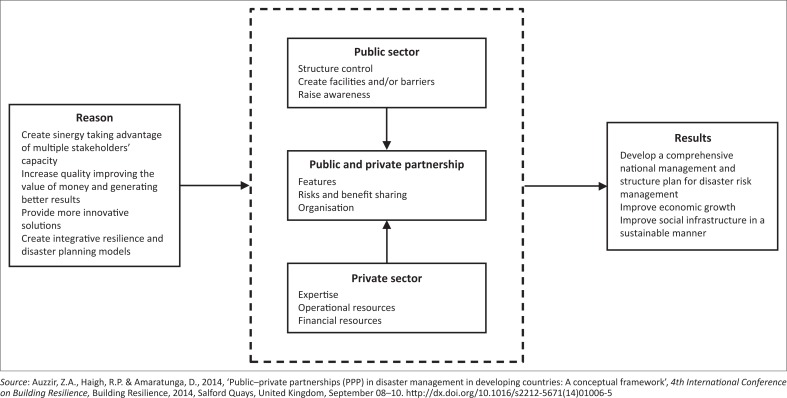
Conceptual framework for public–private partnership in disasters management.

Figure 1 shows a conceptual framework for PPP in disaster management developed by Auzzir *et al*. (2014).

To a certain extent, Figure 1 summarises the theoretical discussion regarding PPP in DRM. However, there are many challenges for the actualisation of PPP in DRM, especially those aspects related to the exchange of knowledge and learning. Partners must build relationships bearing in mind that there are many forms of learning and that benefits tend to emerge during the process, thus potentially improving agility, adaptability and alignment in the supply chains in humanitarian operations (Lima & Gonçalves 2011).

Over the past three decades, the concepts of interorganisational, organisational and interpersonal trust have been extensively studied from a number of perspectives, including economic, psychological and social aspects (Kovács & Tathan 2010). These are, among others, some of the topics to be addressed in partnership formation. Stewart, Kolluru and Smith (2009) use PPP to improve community resilience in disaster situations and identify opportunities to improve resistance following catastrophes.

However, PPP for DRM also presents challenges in its coordination. Some of these challenges and their solutions are shown in Table 1.

**TABLE 1:** Challenges and alliances.

Challenges	Solutions
Lack of mutual understanding	To specify the necessities as soon as participation channels are prede-fined so that expectations are met when and where needed.
Lack of transparency and responsibility	To agree on communication strategies to avoid conflicting messages that may compromise the partnership validity.
Commitment level	To develop engagement rules that define needs in advance and that can be fulfilled by the alliance, together with protocols and guidelines to reach agreement on service levels and clarify expectations of different levels and stages.
Roles and responsibilities	To determine areas to improve skills and allow each party to focus on areas where they can best contribute.
Relations management	To develop partnerships in non-emergency periods. Building relationship and getting to know each other requires significant investment from both sides.

*Source*: Adapted from Tomasini, R. & Van Wassenhove, L., 2009, ‘!Humanitarian logistics. Insead Business Press WFP’, viewed 27 March 2015, from http://www.wfp.org/how-to-help/companies/partner/tnt

In this regard, some successful partnerships are described, such as that of *Thomas Nationwide Transport* (TNT), a world leader express delivery company that has developed a coordination model to create partnership with the humanitarian agency *World Food Programme* (WFP). Following this model, they have succeeded in overcoming barriers and finding appropriate solutions by clearly defining roles and responsibilities in order to make the partnership work in the long term and by using learning as a central element of this partnership (Tomasini & Van Wassenhose 2009).

Margulescu and Margulescu (2013), for example, support the prevention risk management strategy by using an index-based insurance cover in which the risk of natural catastrophe is transferred to the private sector.

Rakesh and Sriparna (2014) present a PPP case in India between an ambulance service company in Mumbai, Ziqitza Healthcare Limited (ZHL) and local governments. Specialists recommended PPP modeling as a way to improve services and to expand access to them. Although ZHL founders were initially apprehensive to work with the government, they finally saw it as an opportunity to fulfil their social mission and also to expand.

In Brazil, the partnership between *Federal Express Corporation* (FedEx) and the local government during the disaster that hit the city of Blumenau (Santa Catarina state) in 2008 to a certain extent allowed for information to be shared and logistic solutions to be identified. According to Fonseca *et al*. (2012), these solutions could have been found prior to the event if partnerships had been defined and aligned as to what actions had to be taken in case of disasters. Moreover, partnerships have the potential to improve individual capabilities.

The private sector can help the public sector with resources and expertise in DRM while seeking opportunities to improve its impact on society by means of responsible actions and partnerships that have the potential to exploit both organisations’ core competencies, improve disaster prevention, increase awareness of corporate brand, and contribute to DRM and mitigation (Tomasini & Van Wanssenhose 2009).

With regard to the private sector-risk management relationship, advances in CSR, which can be seen as an opportunity for social engagement, stand out. Van Bellen (2005) points out that society is increasingly more aware of the CSR issue because of the damage caused by corporations. In 1930s in Japan, for example, the city of Minamata suffered severe consequences due to mercury contamination caused by a company called *Chisso*. In Bhopal, India, an industrial and chemical accident occurred in 1984, in which lethal gases leaked from a *Union Carbide Corporation* pesticide plant. In Ukraine, the 1986 catastrophic nuclear accident in the Chernobyl Nuclear power plant hit the top of the International Nuclear Event Scale (INES). In 1989 in Prince William Strait, Alaska, the effects of the *Exxon Valdez* oil leak was a truly ecological disaster. Examples like these encouraged society, from 1990s, to demand for more responsible behaviour (Cortez 2013).

According to Carroll (1991), CSR usually amounts to the fulfilment of economic, legal, ethical and philanthropic responsibilities, that is, companies should be good corporate citizens. For Leandro and Rebelo (2011), CSR is understood as a set of policies and practices which are aligned with the company’s strategic objectives in response to market and community internal demands, based on common interest, generating value for all its stakeholders. Tachizawa (2014) in turn adds that CSR is becoming a parameter and benchmark for excellence for both business and corporate worlds.

Lang *et al*. (2012) observe that although studies on CSR deal with similar issues and complement each other, they are presented under different concepts such as: (1) business ethics; (2) sustainability; (3) stakeholder management; (4) corporate citizenship and (5) CSR. In order to systematise such a diversity of concepts, Schwartz and Carroll (2007) developed an integrative model called value, balance and accountability (VBA), which sums up the five concepts in three constructs that are common to all of them, namely VBA.

According to Schwartz and Carroll (2007), *value* refers to the efficient supply of goods and services that avoids negative externalities as it is the company’s obligation to generate net societal value, which occurs when the organisation’s objectives and society’s interests are met. With *balance*, the objective is to meet stakeholders’ conflicting interests, including non-stakeholders’, such as the environment and moral standards. This construct represents the process in the VBA model and everyone in the company should actively contribute to its realisation. As for *accountability*, which also means transparency, it refers to the organisation’s duty to engage itself in a correct, sufficient, propitious and verifiable process of observance of those activities that may affect other individuals. This component represents the principles of the VBA model.

In such a scenario, it is necessary to mention the importance that the CSR theme has on developing concepts to attend disaster risks in order to work the partnership between public and private organisations. Hoeffler and Keller (2002), Du, Bhattacharya and Sen (2011), and Garrido, Cunha and Cavalcante (2014) stress the need for governments to be interested in companies’ social actions and suggest that public policies should encourage private companies CSR.

### Disaster risk management

DRM is a management tool that in an interinstitutional manner allows eliminating or reducing risk factors, manages adverse events when they occur in a vulnerable environment and also restores this environment. To Lapolli (2013), DRM aims to invest both organisations and the population with response and resilience capacity in order to get as little material and human damage and loss as possible.

Pietro (2007) takes the issue further and suggests that DRM should be considered as a strategy that benefits interdisciplinary and multisectoral behaviour. Moreover, it should be viewed as a collective attitude rather than an activity.

**FIGURE 2:**
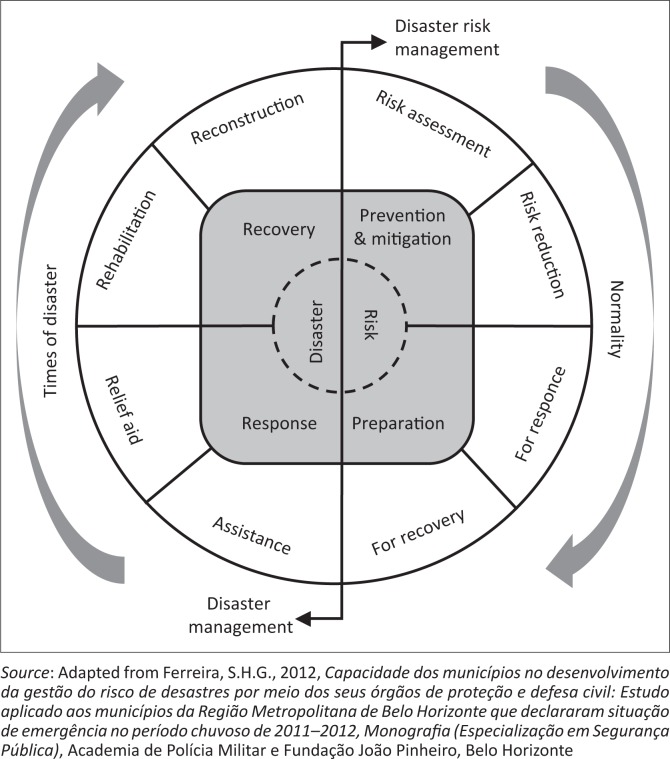
Disaster risk management process.

The DRM process includes prevention, mitigation, preparation, response and recovery actions aimed at the safety of the population in disaster circumstances. Figure 2 shows the complete DRM process.

It can be observed that in times of normality (DRM) and in times of disaster (disaster management) there is great diversity of activities that allow intersectoral and community collaboration under local coordination.

Risk management in turn should enable the creation and implementation of policies, strategies, programmes or actions to reduce the risks for which there must be: (1) clear role definition; (2) functions and responsibilities; (3) the adoption of a decentralised model of power and decision; (4) use of existing structures and organisations, as well as the acknowledgement of coping actions already in place, which should be carried out from a sustainable process and finally, (5) implementation of joint actions with possibility of continuity (Furtado 2012).

Over the past decade the paradigm of public administration has gained strength. This paradigm brings some important elements for DRM, especially social participation in decision-making processes in public affairs. In governance, the perspective of the state-society relationship changes, shifting from vertical relations to horizontal participatory relations. The state continues with its role as advisor and supporter but allows social participation in the decision-making processes, incorporating specific mechanisms such as participatory democracy in order to obtain greater collaboration (Plata 2011).

Collaboration and coordination in DRM favour resilience of systems exposed to adverse events. According to Cardona (2012:15), ‘resilience is the ability to absorb or resist potential impacts generated by the occurrence of a natural event’. Resilience depends on the social system organisation to increase its capacity to learn with previous disasters in order to be better protected in the future and to improve risk-reduction measures (EIRD/UNISDR 2004).

It is important that the DRM process be based on principles especially created to guide it. The 1991 and 2004 UN General Assembly Resolution 46/182 and 58/114 (UN/AGONU), respectively, endorse the fundamental principles in humanitarian assistance: (1) humanity (human suffering should be alleviated wherever it exists); (2) neutrality (the humanitarian agents must not take sides in hostile situations or engage in controversies of political, racial, religious or ideological nature); (3) impartiality (humanitarian assistance should be simply provided as needed, prioritising the most urgent cases of distress, without making any distinction) and (4) independence (it deals with the autonomy and independence that humanitarian action must keep from political, economic, military and other objectives).

The Sendai framework 2015–2030 is one of the benchmarks for DRM. Its objective is to achieve, within the next decade, a substantial reduction of disaster risk, loss of lives, means of subsistence and health, as well as individuals’, companies’, communities’ and countries’ economic, physical, social, cultural and environmental assets. Furthermore, it aims at conducting disaster management from a multi-risk and multisectoral approach, covering risks of any intensity, frequency, sudden or of slow evolution and of various origins (UNISDR 2015).

According to UNISDR (2015), disaster risk reduction requires shared responsibilities by central governments, local authorities and other sectors and stakeholders, even though DRM must protect people and their property, their livelihoods and productive assets, including the right to development. Thus, the process requires the whole society’s commitment and partnership, in a clear designation of responsibilities between public and private stakeholders.

According to UNISDR (2015) the new Sendai framework outlines four priorities for action: (1) understanding disaster risk; (2) strengthening disaster risk governance to manage disaster risk; (3) investing in disaster risk reduction for resilience and (4) strengthening the disaster preparedness for effective response, and to ‘Build Back Better‘ in recovery, rehabilitation and reconstruction. The priorities for action highlight the need for planning, based on current sustainable development paradigm that requires everybody’s participation.

Local development can be understood by the harmonious progress between the population’s welfare and land usage, conservation and protection of natural resources, and productive activities with the objective of improving population’s quality of life in a sustainability approach, which in turn is associated with the performance of sectors, such as public, private and community (Secretaria General Da Comunidade Andina [SGCA] 2009).

Hewitt (2013) draws attention to the existing relation between disasters and communities suffering from the lack of development. Communities’ development is related to the style and direction adopted, and it requires protection actions that can alter or redistribute risk to effectively obtain sustainable development in all dimensions.

## Method and development

**FIGURE 3:**
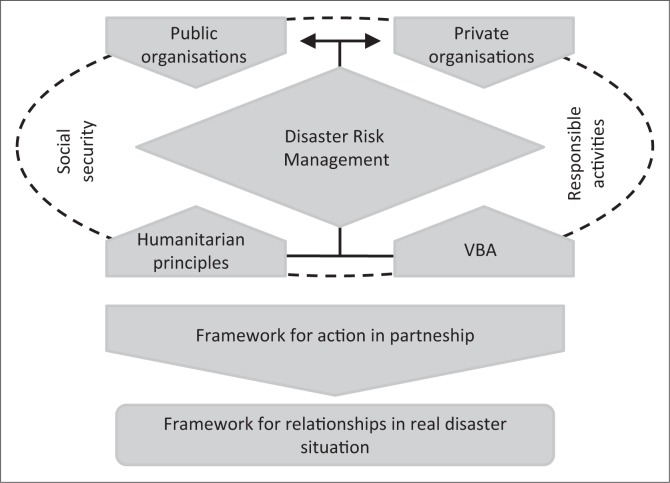
General scheme of study.

Different organisations’ motivation for the involvement in DRM starts from distinct principles. Figure 3 shows the DRM partnership context, as well as the study contribution perspective.

Based on this context, we propose a framework for action for the implementation of PPP in DRM. Public organisations are responsible for social security, for which they must follow humanitarian principles for DRM. With regard to private organisations, they can collaborate, motivated by the principles of CSR, and consequently through responsible activities and actions assist in the process of risk and internal disaster management such as that of the local system.

The proposal is based on the new Sendai framework 2015–2030 priorities and actions, especially those that allow public and private collaboration at local level. For example, for *priority 1*, *understanding disaster risk*, we used the action that deals with the knowledge and training on reducing the risk of disasters of all sectors. As the framework is based on literature review, empirical data are integrated to evaluate the relationships with a real situation, which is discussed in the *Experience from the disaster in Xanxerê – Santa Catarina* section below. For such, a visit was held to the severely tornado-hit city of Xanxerê, in Santa Catarina (SC) state. Interviews were held with the representative of the Municipal Civil Defence (Coordenação Municipal de Proteção e Defesa Civil) and the representative of the local business association. Improvements to the framework were proposed based on the qualitative analysis of data obtained from the interviews and experience.

The next sections are organised as follows: development of framework for action in the creation of PPP, description of the adverse event which occurred in the city of Xanxerê and finally, analysis based on action proposed in the framework related to the described adverse event.

### Framework for actions

Before presenting the framework for actions, we wish to stress the importance of establishing minimal criteria to be met by partners for each proposed action. Hall (1984) deals with interorganisational relations for the set of actions that consist of a group of organisations that form a temporary alliance for a limited goal that may have their agreements formalised, their work shared, their behavioural norms defined, and new member recruitment based on well-defined principles. Tomasini and Van Wassenhove (2009), based on the experience of the partnership between TNT and WFP, point out that the search for focus, reputation, neutrality and balance of criteria such as skills, public relations, effectiveness and geographical scope is one of the main actions in the search for humanitarian organisation partnership.

In view of such a scenario, Table 2 presents the proposed actions, which are at first described (1) based on the Sendai framework. We suggest, respectively, how the public (2) and private (3) sectors should act to achieve the proposed action, and finally, we describe how these actions can be realised with the partnership (4).

**TABLE 2:** Framework of proposed actions for public and private partnership in disaster risk man-agement based on the Xanxerê case study in Brazil.

(1) Actions based on the Sendai framework	(2) Public sector	(3) Private sector	(4) Proposed action with the partnership
Raise awareness for the involve-ment between DRM with public and private organisations and the community.	Weigh losses in recent years in the community regarding infra-structure and public services; start awareness campaigns from identified losses; provide events for the whole city in order to clarify DRM with population responsibilities in general; de-velop disaster risk resilience in public workplaces through struc-tural (infrastructure) and non-structural (awareness) meas-ures.	Weigh direct and indirect losses in companies in recent years related to disasters; promote business association encouraging compa-nies involvement with DRM, including bringing CSR concepts; develop disaster risk resilience in the workplace through structural (infrastructure) and non-structural (awareness) measures; and ensure continuity of services and the integration of DRM in models and business practices.	Initiatives to awareness level should be integrated and, in order to be effective, they need an open and constant communication channel between the partners. Public sector should promote the private sector qualification in DRM and its application in the local context and at business level. Private sector in turn should financially support and disseminate the public sector’s initiatives and campaigns.
Include DRM subject in vocational education and training.	Include DRM design in school curriculum and empower teach-ers; determine risk multipliers and disaster management knowledge; deliver lectures or trainings or workshops in educational institu-tions at all levels; and spread knowledge of DRM to community and private organisations.	Financially support the hiring of professionals to conduct lectures or trainings or workshops and/or make them available if they are part of the staff; suggest and support the qualifications of those responsible for DRM and support community work; and encourage, organise working teams to participate in DRM training.	Knowledge of DRM should be a priority in order to increase community resilience. Public sector should share knowledge and prepare teachers and multi-pliers. In addition to creating internal risk management pro-grammes, private sector can provide team members to take part in training and support for activities on management of risks and local disasters.
Local collaboration in disseminating information on the risks of disaster.	Provide information from various sources; pass on information to the press in organised fashion; provide training to targeted media for DRM communication.	Allow press professionals and companies with communication departments to take part in training for communication on DRM, and inform population and/or employees about the risks of a disaster aiming at reducing it and increasing resilience in times of normality and in times of disaster.	Public sector should guide com-munication for periods of normal-ity and times of disaster, as well as maintain a steady flow of infor-mation to the media. Private sector should support the wide dissemination of information and adhere to the regulations that apply to communication (public press) and communication de-partments for internal communi-cation. In such a partnership, DRM gains visibility and communication becomes a fundamental vector for community orientation.
Promote local sustainable devel-opment that stems from internal and external integration with ampler coordination, which influences the development of disaster risk reduction view in different ways.	Develop local risk management plan by observing sustainability in all its dimensions, involving the private sector in all discussions; integrate disaster risk assess-ments on implementation and development of land use policies, preserving business and protect-ing livelihoods and the production of goods along the supply chain; set goals, strategies, indicators and targets in order to eliminate the creation of risk and reduce existing risk; promote qualification and selection of private organisations in accordance with their needs ; and integrate risk management plan to municipal management plan.	Participate in the planning and review stages adding value and knowledge; propose and carry out performance schedule based on principles of value, balance and transparency in business; take an active role in disaster risk reduc-tion within the business activities framework as well as in part-ner’s awareness and employees’ conduct; and based on the worldwide appeal for the private sector involvement in managing disaster and risks, assume the roles and responsibilities in accordance with collaborative capabilities.	The management plan led by the public sector with ample partici-pation of the private sector and the community allows strategic objective definition and the allocation of roles and responsi-bilities for those involved. The private sector directly gains by supporting the planning with the transfer of knowledge and infor-mation, as it may contribute to local development and conse-quently thrive in their businesses with a view to corporate social responsibility.
Promote mechanisms for disaster risk transfer to mitigate the impacts of disasters on local economy.	Promote the need for security and protect public and private property in order to reduce dis-asters financial impact on gov-ernments and societies in urban and rural areas.	Provide insurance cover based on local need (insurance companies or local representatives); and share experiences on risk trans-fers of private initiative with the local coordination and DRM.	This action allows discussion between public and private sec-tors in the search for alternatives and makes them available, in addition to promote better guid-ance to the public on the opera-tion in order to mitigate the impacts of disasters.
Promote continuously disaster preparedness with response exer-cises and recovery, including evacuation tasks, training and the establishment of area-based sup-port systems in order to ensure a rapid and effective response to disasters and related displacement, including access to safe shelter, food and non-food supplies.	Develop contingency plans for local disaster risks; research about needed and available resources, and also about what should be sought from the private sector; organise an exercise schedule including the entire population; transfer disaster management knowledge by means of volun-teers and corporate team training; and strengthen partnerships with private organisations’ technical and logistical answer capacities.	Participate in contingency plans for disaster risks preparation; provide infrastructure as well as products and services assuming protocol commitments; effectively participate in preparation, allowing and encouraging volunteering and training of internal teams able to operate in adverse events; share technical and logistic private knowledge to provide emergency public service; and financially support preparation exercises.	In this action, the knowledge sharing, as well as human and material resources, is critical. Disaster generates losses for both sectors; therefore, preparation benefits all parties.
Promoting cooperation of various institutions, various authorities and related agents at all levels, including affected communities and busi-nesses.	Allow private sector participation by sharing power and decision on the structure of DRM; create a continuous communication sys-tem with those involved; promote the development of quality stan-dards, such as certifications and awards for DRM; and encourage cooperation through the promo-tion of qualified companies in DRM.	Encourage and financially support initiatives of coordination involv-ing local DRM; promote internal initiatives for resilience develop-ment; develop innovative in progress projects, roads and others that represent threats to its users; and participate through business association in the de-velopment of quality standards, such as certifications and awards for DRM.	A look at prevention rather than just the answer and ample public authority, private sector and community participation are perhaps the largest current para-digms for DRM.

DRM, disaster risk management; CSR corporate and social responsibility.

We hope that research communities feel encouraged to contribute with the initiatives proposed in Table 2, either from a theoretical or practical perspective.

### Experience from the disaster in Xanxerê – Santa Catarina

SC is one of the 26 Brazilian states. Located in the southern region of the country, it has 295 municipalities and a population of 6 248 436 according to the Brazilian Institute of Geography and Statistics (IBGE) (2013). The state has been hit by floods, abrupt and gradual climate changes, landslides, droughts, tornadoes, fogs and storms. In 20 April 2015, the city of Xanxerê, located in the West of SC, was hit by a tornado with winds of over 200 km/h.

The city has a population of 47 600 and it had entire neighbourhoods and central regions destroyed. The tornado left 539 people homeless, and 4275 people had to be relocated. Additionally, 4 people died and 97 were injured. More than 2000 public, commercial and residential buildings were hit. According to the Civil Defence Secretary, the severe weather caused economic losses of more than 49 million to residential buildings, of more than 45 million to commercial buildings, and next to 10 million to public buildings (SDC/SC 2015).

The Civil Defence Secretary provided roof tiles, protective canvas, food items and cleaning kits (SDC/SC 2015). The local government has no exact idea of the donations received and to whom they were handed out. However, it claims that there was substantial donation, even though they were still in need of some basic materials. Response and recovery involved more than a thousand professionals and more than 200 vehicles. This does not include volunteers and private organisations that worked independently.

### Analysis relating the proposed framework for actions with the event in Xanxerê-SC

The relation between the proposed framework for actions (PFA) and the adverse event were established from interviews with experts who attended at the aftermath of this event. On the public sector side, the head of the Civil Defence administration (COMPDEC) was interviewed, and on the private sector side, the head of Xanxerê Business Association (ACIX). We observed the PPP relation from the experience with the tornado in Xanxerê and we were able to infer important aspects from the PFA.

In general, ACIX believes that its staff and the management need to obtain the same level of understanding about DRM in order to timely support the public sector in disseminating information and starting up campaigns. Considering CSR practices to the point of allowing PPP, ACIX understands that it needs a substantial amount of information and, together with the public sector, it needs to know how to carry out proper management. Based on VBA, ACIX believes that it is not possible to generalise the practices adopted by Xanxerê’s companies; however, when the disaster occurred every company helped as well as they could.

From COMPDEC’s point of view, PPPs could occur through exchange, in which those responsible for DRM could take prevention knowledge to companies who, in turn, could contribute with DRM funding. The need for businesses’ awareness in supporting civil defence and actualising internal emergency services must be strengthened. With regard to *VBA*, companies need to change their view and expect less from the government.

With regard to humanitarian principles, which are internationally recognised for action in response and recovery operations in disasters, according to COMPDEC, haste, nervousness and fatigue made things go undetected. However, they tried to attend to all in the best possible manner, even though all decisions were taken jointly with the public ministry’s guidance. For ACIX, the principles were observed, with some aspects to be improved on as, with the exception of the Civil Defence’s agents, there was no response and recovery preparation.

As for the proposed framework, according to ACIX, the weighing of both public and private sector losses is essential for starting awareness campaigns. The companies’ direct and indirect losses were substantial, especially due to the delay in reconstruction. According to COMPDEC more than 100 companies were affected, some of which will not be able to restart their activities. Furthermore, it indicates that businesses’ involvement in preventing may allow preparation for disaster.

ACIX pointed out the importance of both the Association and businessmen acquiring DRM knowledge. As for the proposal of local collaborative action to disseminate information on disaster risk, one of the aspects that have been observed is the lack of continuous communication between those responsible for DRM and the private sector. Thus, ACIX suggests the production of a booklet with rights and duties indicating both public and private bodies’, as well as the population’s responsibilities.

Still with regard to the framework, COMDEC indicated that many of those affected did not obtain information on insurance. ACIX believes that government-subsidised credit should be made available as early as possible in case of disasters such as the one occurred in Xanxerê.

COMDEC also points out that at the time of the disaster companies assisted with labour force, lorries and power generators, among others items. However, the major difficulty is to establish permanent partnerships. For ACIX, cooperation took place partially in the private sector, taking into account that who could help physically and financially did so during response and recovery. With regard to planning for sustainability, it was observed that there is no initiative involving corporate bodies in the city.

There are many challenges for partnership actualisation; however, communication strategies should avoid conflicting messages that might compromise their validity. Moreover, with regard to the level of commitment, it is important to develop engagement rules that define needs in advance by discussing protocols and guidelines in order to define service levels, roles and responsibilities, as well to determine areas to improve skills and allow each party to focus on the areas in which they can best contribute and strengthen relation management.

Summing up, the analysis of the proposed framework in relation to the Xanxerê-SC experience reinforces the need for wider discussion of collaborative processes in DRM. Data reveal that there were no cooperative initiatives between private and public sectors prior to the adverse event, although the parties are aware of the need for a change in scenario. Despite the lack of clarity regarding how changes can be promoted, both organisations agree that the PFA on partnership is feasible through a shift in paradigm in both sectors.

## Conclusion

In this article we presented our PFA for partnership formation considering objectives for action and signs of collaboration and coordination relationships, linking them to the adverse event that occurred in a city of Xanxerê in the state of Santa Catarina in April 2015. Thus, several key concepts were discussed, such as *CSR*, *VBA*, *Partnership* and *DRM*.

By analysing the actions in the PFA for the disaster that occurred in Xanxerê, it was observed that there is a need for private organisations’ involvement with public organisations and vice versa. The partnership is necessary in order to raise awareness for more involvement in DRM between organisations and communities, to allow exchange of information, to facilitate communication between public and private organisations, and above all to allow exchange of knowledge. PPP can help municipalities and regions increase their resilience capacity to face crises and return to their activities in disaster situations.

With such an approach, it is possible to identify a correspondence between private companies’ competencies and activities with public organisations. In general, based on Tomasini and Van Wassenhove (2009) and *VBA* concepts, while operational assistance activities in an emergency crisis may remain under the care of the humanitarian organisations, since dealing with unforeseen circumstances is their core competency, the private sector can supply affordable and professional services. And at the preparatory stage, when alignment occurs, private sector is able to transfer knowledge and experience to humanitarians.

The studied empirical data confirm the initial need of effective governance and DRM on site that allow strategic definition, roles assignment, and responsibilities for those involved in the process. VBA is considered an alternative for private organisations’ involvement through PPPs in DRM. However, for improved governance, new PPPs and ample use of VBA concepts requires stakeholders’ training.

Finally, we draw attention to the fact that SC is considered a tornado corridor that has been hit in various regions by approximately 80 tornadoes over the past four decades. However, despite such a threat, among others, nothing has raised enough attention from either public managers, private initiative or the community as a whole to effective governance in DRM. We hope that this study encourages further studies in order to broaden both theoretical and practical DRM perspective.
